# Are Proactive and Reactive Aggression Meaningful Distinctions in Adolescents? A Variable- and Person-Based Approach

**DOI:** 10.1007/s10802-016-0149-5

**Published:** 2016-04-26

**Authors:** K. C. Smeets, S. Oostermeijer, M. Lappenschaar, M. Cohn, J. M. J. van der Meer, A. Popma, L. M. C. Jansen, N. N. J. Rommelse, F. E. Scheepers, J. K. Buitelaar

**Affiliations:** 1 Karakter Child and Adolescent Psychiatry, Reinier Postlaan 12, 6525 GC Nijmegen, Netherlands; 2 Child and Adolescent Psychiatry, VU University Medical Center Amsterdam, Amsterdam, Netherlands; 3 Donders Institute for Brain, Cognition and Behavior, Department of Cognitive Neuroscience, Radboud University Medical Centre, Nijmegen, Netherlands; 4 Radboud University, Nijmegen, Netherlands; 5 Rudolf Magnus Institute of Neuroscience, Department of Psychiatry, UMC Utrecht, Utrecht, Netherlands; 6 Donders Institute for Brain, Cognition and Behavior, Department of Psychiatry, Radboud University Medical Centre, Nijmegen, Netherlands

**Keywords:** Proactive and reactive aggression, Latent class analysis, Factor analysis, Adolescents

## Abstract

This study was designed to examine whether proactive and reactive aggression are meaningful distinctions at the variable- and person-based level, and to determine their associated behavioral profiles. Data from 587 adolescents (mean age 15.6; 71.6 % male) from clinical samples of four different sites with differing levels of aggression problems were analyzed. A multi-level Latent Class Analysis (LCA) was conducted to identify classes of individuals (person-based) with similar aggression profiles based on factor scores (variable-based) of the Reactive Proactive Questionnaire (RPQ) scored by self-report. Associations were examined between aggression factors and classes, and externalizing and internalizing problem behavior scales by parent report (CBCL) and self-report (YSR). Factor-analyses yielded a three factor solution: 1) proactive aggression, 2) reactive aggression due to internal frustration, and 3) reactive aggression due to external provocation. All three factors showed moderate to high correlations. Four classes were detected that mainly differed quantitatively (no ‘proactive-only’ class present), yet also qualitatively when age was taken into account, with reactive aggression becoming more severe with age in the highest affected class yet diminishing with age in the other classes. Findings were robust across the four samples. Multiple regression analyses showed that ‘reactive aggression due to internal frustration’ was the strongest predictor of YSR and CBCL internalizing problems. However, results showed moderate to high overlap between all three factors. Aggressive behavior can be distinguished psychometrically into three factors in a clinical sample, with some differential associations. However, the clinical relevance of these findings is challenged by the person-based analysis showing proactive and reactive aggression are mainly driven by aggression severity.

**Electronic supplementary material:**

The online version of this article (doi:10.1007/s10802-016-0149-5) contains supplementary material, which is available to authorized users.

Aggression can be defined as behavior directed at an object, human or animal, which causes harm or damage (Bushman and Anderson 2001; Gannon et al. 2007), and is one of the most frequent reasons for referral of children and adolescents to mental health services (Armbruster et al. 2004; Rutter et al. 2010). Aggression is assumed to be a heterogeneous construct, and a distinction is often made between two different subtypes, reactive and proactive aggression. Reactive aggression refers to an emotionally charged response to provocations or frustration and is also known as “impulsive”, “hot blooded” or “affective” aggression (Dodge and Coie 1987; Kockler et al. 2006; Stanford et al. 2003). Proactive aggression refers to a conscious and planned act, used for personal gain or egocentric motives, also known as “premeditated”, “instrumental” or “cold-blooded” aggression (Blair et al. 2006; Blair 2001; Dodge and Coie 1987; Frick and Ellis 1999).

Support for the distinction between proactive and reactive aggression is provided by several *variable-based studies* (using factor analysis and correlations) in clinical and nonclinical samples of adolescents and adults (Cima et al. 2013; Dodge and Coie 1987; Raine et al. 2006). Furthermore, these subtypes of aggression have been related to distinct behavioral, neurocognitive and treatment profiles (Card and Little 2006; Polman et al. 2007). Reactive aggression is associated with attention problems, anxiety problems, peer rejection, hostile attribution bias, emotional dysregulation, deficits in problem solving, low verbal intelligence, and often appears earlier in life than proactive aggression. In contrast, proactive aggression is related to delinquent behaviour, lower levels of victimization, positive outcome expectancies, and higher self-efficacy about aggression (Blair 2013; Cima and Raine 2009; Dodge and Coie 1987; Merk et al. 2007; Vitaro et al. 2006). Moreover, different neural mechanisms have been suggested to underlie reactive and proactive aggression. Reactive aggression has been linked to hypofunction of in particular the orbitofrontal and anterior cingulate cortex, and increased responsiveness of the amygdala to distress, whereas proactive aggression has been associated with dysfunction of the ventromedial prefrontal cortex and the striatum, and decreased responsiveness of the amygdala to distress (Blair et al. 2006; Blair 2013).

However, other data challenge the assertion that proactive and reactive aggression can be regarded as distinct constructs. Systematic reviews report that proactive and reactive aggression correlate highly (up to *r* = 0.87); (Card and Little 2006; Polman et al. 2007). Furthermore, most studies used partial correlations and corrected for shared variance, which makes it unclear which part of the variance was examined and whether shared or independent associations were shown (Lynam et al. 2006). This suggests that aggression is one construct which cannot be subdivided in different subtypes.

An untouched aspect of this variable-based approach is whether a distinction between reactive and proactive aggression can be made at the level of the individual (*person-based approach*). In other words, can we reliably distinguish individuals predominantly showing reactive aggression from those predominantly showing proactive aggression? Previous research shows that identifying distinct correlations using variable-based methods cannot necessarily be clearly translated to clinical characteristics within a person (Crapanzano et al. 2010). Therefore, results of methods using multiple regression procedures may appear not significant in one person or are misleading, due to the absence of a proactive-only group or to overlapping constructs (Crapanzano et al. 2010). Previous research has suggested that proactive and reactive aggression tend to co-occur in the same individuals, with only a small proportion of clinically referred children and adolescents presenting with proactive aggression only (Barker et al. 2006; Barker et al. 2010; Kempes et al. 2005). Furthermore, research shows that informants (teachers, parents or peers) find it hard to observe and identify the distinction between proactive or reactive aggression (Kempes et al. 2005). Therefore, it may be questioned whether the distinction holds in clinical practice.

To the best of our knowledge, no other studies have combined a variable-based and person-based approach regarding the subtypes of aggression, while it has been shown efficient to combine both methods to explore the underlying latent structures of psychological constructs (Masyn et al. 2010). This combination is highly relevant for clinical practice, since assessment and treatment decisions are made at the level of the individual (person-based approach), rather than at the variable or factor level, warranting investigation of their relation.

We hypothesized that proactive and reactive aggression would be found as distinct factors in the variable-based analysis. Second, we hypothesized that the person-based analysis would yield different classes of individuals including the presence of both subtypes in the individual and reactive or proactive aggression with the absence of the other subtype (Kempes et al. 2005). We further anticipated that proactive and reactive aggression, both at the variable and at the person level, would differ regarding their associated behavioral correlates. We expected that reactive aggression would be particularly associated with anxiety and attention problems, and proactive aggression with increased levels of conduct disorder symptoms (Vitaro et al. 2002). These aims were examined in 587 adolescents (mean age 15.6; 71.6 % male) from clinical samples of four different sites.

## Methods

### Participants

We were able to aggregate data from 587 adolescents who were referred to clinical practice because of their externalizing behavior problems from four Dutch studies. The four clinical subsamples were selected to capture the entire adolescent aggression range and differed with respect to the mean study aggression level: 131 participants from a special school for children with disruptive behavioral problems, 199 participants from a residential facility for treatment of conduct problems, 154 adolescents with a history of being arrested by the police before the age of 12 (Cohn et al. 2015; Domburgh et al. 2009), and 103 participants from a Dutch diversion program for delinquent youth (Popma et al. 2007). Participants of the first two studies were asked to fill in questionnaires at the start of their treatment within the school program or within the residential facility. Adolescents being arrested before the age of 12 were asked to fill in questionnaires at follow-up, approximately 5 years later. Furthermore, adolescents from the Dutch diversion program completed questionnaires between 4 to 10 weeks after they were referred to the diversion program for committing a minor offense. All participants were between 12 and 20 years old (*M* = 15.6, *SD* = 1.9), 51.4 % were of non-Caucasian origin, 71.6 % were male and the sample was characterized by an IQ in the average range (*M* = 88.3, *SD* = 15.3; IQ based on Wechsler Intelligence Scale- III). Written informed consent was obtained from every participant and parents or caregiver before enrolment in the study and all studies were approved by the local ethical committees. All participants (except subjects within the residential facility) received a small financial reimbursement after completing several questionnaires.

### Measures

#### Reactive-Proactive Aggression Questionnaire (Self-Report)

The Reactive-Proactive Aggression Questionnaire (RPQ) was used to assess proactive and reactive aggression (Raine et al. 2006). The RPQ is a 23-item self-report questionnaire with a 3-point Likert scale, assessing the frequency at which participants have engaged in the type of behavior described in each item, as follows: *never* (0), *sometimes* (1) or *often* (2). The instrument yields a total score for aggression and scores for two subscales: proactive aggression (12 items) and reactive aggression (11 items). These subscales represent a 2-factor model with acceptable fit indices, based on data from the USA (Raine et al. 2006). The questionnaire was back-translated by a native English speaker.

#### Youth Self Report and Child Behavior Checklist (Self-Report and Parent-Report)

The Youth Self Report (YSR, official Dutch version 1999/2001) (self-report, 11–18 years) and Child Behavior Checklist (CBCL, official Dutch version 2001) (parent-report, 6–18 years) were used to specify the behavior profiles of the observed classes (Achenbach et al. 2008). These questionnaires are widely used for the assessment of behavioral problems in children and adolescents. They comprise over one hundred items, rated on a 3-point scale of *not true* (0) *somewhat or sometimes true* (1) or *very or often true* (2). The instruments yield eight syndrome subscales and six DSM related subscales. In this study only the DSM related scales, and total internalizing, externalizing and overall total subscales were used (see Table 5), (Achenbach et al. 2008). The total scores of the subscales are converted into T-scores with a cut-off score (based on behavioral profiles of a healthy norm sample) that shows the severity of the problems (subclinical or clinical levels). Good reliability (Cronbach’s alpha’s ranging from 0.79 to 0.95 for all the scales) of the CBCL and YSR has been reported (Achenbach et al. 2008). Also, the CBCL and YSR scales of the current study showed acceptable to good reliability (Cronbach’s alpha between 0.70 and 0.88 for CBCL scales and 0.76–0.84 on YSR scales. Only YSR anxiety showed a Cronbach’s alpha of 0.65). For the participants recruited from the school in Rotterdam a previous version of the YSR (1991) was used (Achenbach et al. 2008). In the new version (2001), the content of items 2, 4, 5, 28, 78 and 99 differ from the content of the items in the old version. This was solved by using the mean score of the subscale (leaving the old items out) multiplied by the number of actual items on that subscale. No YSR and CBCL data were available for the participants of the Dutch diversion program.

#### Procedures

Participants in this study had been referred to special schools or clinical services because of externalizing behavior, and/or had been arrested. If informed consent was given, participants completed the RPQ and YSR questionnaire, while their current primary caretakers completed the CBCL.

#### Data Analyses

First, we checked the two factor model of the RPQ (Raine et al. 2006), using Confirmatory Factor Analysis (CFA), and conducted Exploratory Factor Analysis (EFA) to examine alternative factor solutions of the 23-items of the RPQ using Mplus 6.11. Second, a multi-level Latent Class Analysis (LCA) was conducted, which takes into account within-center measurement bias, by using the factor solution of the RPQ and different sites as input. LCA is a cluster analysis, used to identify homogeneous classes of subjects with similar profiles of aggression in the observed data (Supplement S1). Third, we calculated Pearson’s correlations between the RPQ aggression factors found in the factor analyses and the DSM-based and total internalizing and externalizing behavior scales of the YSR and CBCL, to examine whether the aggression factors showed differential relationships with other domains of psychopathology. We examined whether significant differences between the correlations of the three factors and the YSR/CBCL subscales were present using transformation into z-scores (Lee and Preacher 2013). Finally, the latent classes were characterized in terms of RPQ factor scores and psychopathology as indexed by the YSR and CBCL by dependent t-tests (within-class comparison) and MANOVA analyses (between-class comparison) with Bonferroni post-hoc tests in SPSS 20.0. Age, gender, IQ and ethnicity were included as covariates and their moderator effects were examined. Partial eta squared was used to define the effect size (0.01 to 0.06 small, 0.06 to 0.14 medium and 0.14 or higher large effect size (Cohen 1988). Also, multiple regression analyses were conducted (forward-method) to examine suppressing relationships of the three factors on internalizing and externalizing behavioral scales measured by YSR and CBCL (with age, gender, IQ and ethnicity as covariates), compared to the zero-order correlations.

#### Fit Indices

Results of the EFA were interpreted based on several fit indices, i.e., the Tucker-Lewis Index (TLI; Tucker and Lewis 1973) the Comparative Fit Index (CFI; Bentler 1990) the Root Mean Square Error of approximation (RMSEA; Steiger 1990) and the eigen-value. A TLI or CFI between 0.90 and 1 shows an acceptable to good fit of the model. Also, a RMSEA of 0.06 and lower, and an eigen-value of 1 or higher indicate a good fit of the model (Hu and Bentler 1999). The Bayesian Information Criteria (BIC) (lowest) and Entropy (highest) were used to define the best LCA fit (Nyland et al. 2007).

## Results

### Factor Analyses on RPQ Items

The CFA was conducted to replicate the original 2-factor model of the RPQ (Raine et al. 2006). Fit indices (TLI = 0.954; CFI = 0.958; RMSEA = 0.056–0.066, Estimated RMSEA = 0.061) were acceptable, but did not show a perfect fit (RMSEA <0.06 indicates a good fit). Furthermore, studies of Blair (2013) and Fite et al. (2006) also showed three different subtypes of aggression, suggesting that the three-factor model is likely not an artifact of our sample. Because of these results and no optimal solution of the CFA was found, an EFA was conducted to examine how many factors best described the RPQ (see Table 1 for the fit indices). Results showed a better fit of the three factor model, as compared to the two factor model and four factor model (See Table 1). Moreover, the items of the two factor model from the EFA did not correspond with the items of the original two factor model used in the CFA,

**Table 1:** Fit indices of the confirmatory factor analysis (original 2-factor model) and exploratory factor analysis based on the RPQ

Factor structure	TLI	CFI	RMSEA	Estimated RMSEA	Eigen-value
CFA	0.954	0.958	0.056–0.066	0.061	-
EFA					
2-factors	0.958	0.965	0.053–0.063	0.058	1.46
3-factors	0.974	0.981	0.040–0.052	0.046	1.33
4-factors	0.988	0.982	0.032–0.045	0.038	0.891

The three factor model shows the best fit, since the Eigen-value is >1, the RMSEA <0.06 and the TLI and CFI are >0.90. Furthermore, the two factor model did not correspond with the original two factor model as used in the CFA

In the three factor model, the proactive factor was replicated, but the reactive factor was divided into two factors: “reactive aggression due to internal frustration” and “reactive aggression due to external provocation”, respectively. See Table 2 for the factor loadings, with a factor loading of ≥ 0.40 indicating a strong factor loading. Naming the factor “reactive aggression due to internal frustration” was based on the items with the highest factor loadings, like “Become angry or mad when you don’t get your way”; “Gotten angry when frustrated” and “Gotten angry or mad when you lost a game”. Also, a negative association was found with items “Carried a weapon to use in a fight”; “Used force to obtain money or things from others”; “Had a gang fight to be cool”; “Had fights with others to show who was on top”; “Hit others to defend yourself”. This shows this form of aggression to be mainly based on aggression due to inflexibility, being stubborn and internal frustration rather than proactive aggression or external provocation. Furthermore, naming the factor “reactive aggression due to external provocation” was mainly based on the items “Hit others to defend yourself”, “Reacted angrily when provoked by others” or “Gotten angry when others threatened you”. Negative associations were found with items regarding winning a game, inflexibility, making obscene phone calls for fun or the use of force to obtain money. This shows that this form of reactive aggression is mainly based on external provocation and threat rather than the use of aggression for personal gains or aggression due to inflexibility. It is of interest that the items on factor 3 also load on the proactive factor, but do not load or even negatively load on the “reactive aggression due to internal frustration”, like “carried a weapon to use in a fight” or “had fights with others to show who was on top”. These items are all related to threat and provocation in relation to others, and not to inflexibility. The inter-correlations between these three factors were moderate to high (*r*’s between 0.635 and 0.680 with *p*’s < 0.001), indicating that these are distinguishable but strongly correlated dimensions of aggression. The three factors showed good reliability (Cronbach’s alpha = 0.88 (proactive), 0.76 (reactive internal frustration), 0.82 (reactive external provocation).

**Table 2:** Factor loadings of the EFA based on the RPQ questionnaire

RPQ Items and factor loadings	Factor 1 (Proactive aggression)	Factor 2 (Reactive internal frustration)	Factor 3 (Reactive external provocation)	
15: Used force to obtain money or things from others	0.959	−0.205	- 0.045	Factor 1
12: Used physical force to get others to do what you want	0.745	0.077	0.048	Factor 1
23: Yelled at others so they would do things for you	0.715	0.157	0.004	Factor 1
20: Gotten others to gang up on someone else	0.677	0.127	0.002	Factor 1
4: Taken things from other students	0.674	0.071	0.047	Factor 1
18: Made obscene phone calls for fun	0.669	0.031	−0.159	Factor 1
6: Vandalized something for fun	0.634	0.010	0.084	Factor 1
9: Had a gang fight to be cool	0.658	- 0.112	0.230	Factor 1
10: Hurt others to win a game	0.654	0.019	−0.006	Factor 1
17: Threatened and bullied someone	0.592	0.094	0.185	Factor 1
21: Carried a weapon to use in a fight	0.596	−0.183	0.362	Factor 1 > 0.40
2: Had fights with others to show who was on top	0.493	- 0.009	0.406	Factor 1 ➔ in line with the original proactive factor
11: Become angry or mad when you do not get your way	−0.039	0.910	−0.008	Factor 2
5: Gotten angry when frustrated	0.029	0.756	0.095	Factor 2
13: Gotten angry or mad when you lost a game	0.201	0.462	−0.092	Factor 2
1: Yelled at others when they have annoyed you	0.062	0.427	0.362	Factor 2 > 0.40
8: Damaged things because you felt mad	0.296	0.352	0.302	Factor 2 ➔ highest loading but is very low on all the factors
19: Hit others to defend yourself	0.150	0.000	0.727	Factor 3
14: Gotten angry when others threatened you	−0.029	0.190	0.647	Factor 3
22: Gotten angry or mad or hit others when teased	0.176	0.101	0.571	Factor 3
16: Felt better after hitting or yelling at someone	0.369	0.177	0.349	Factor 3 ➔ because this is originally an reactive item, we did not use it on factor 1.
3: Reacted angrily when provoked by others	−0.017	0.421	0.509	Factor 3 ➔ highest loading but is also associated with F2
7: Had temper tantrums	0.100	0.404	0.426	Factor 3 ➔ highest loading but is also associated with F2

### Multi-Level Latent Class Analysis

**Fig. 1:**
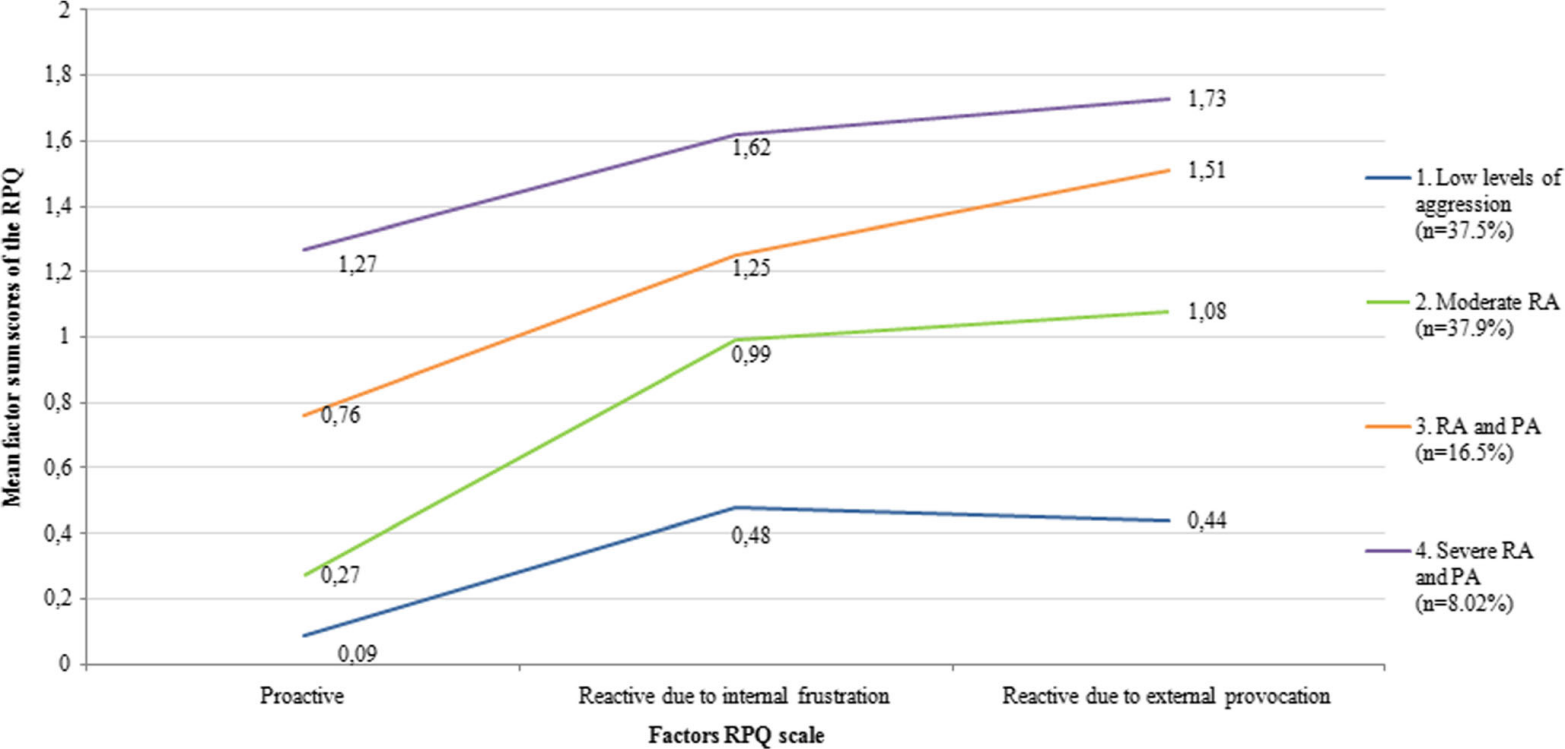
Mean factor sum scores of the RPQ questionnaire per class

The multi-level LCA identified four different classes based on the best fit of the model (BIC) and best fit of each individual into a specific class (Entropy) (Table 3). For the sake of clarity, we labeled the classes as follows. Class 1 is characterized as “Low levels of aggression” (*N* = 220; 37.5 %); Class 2 as “Predominantly reactive aggression/low proactive aggression” (Moderate RA) (*N* = 222; 37.8 %); Class 3 as “Proactive and reactive aggression (higher provocation induced aggression compared to frustration-induced aggression)” (PA & RA) (*N* = 97; 16.5 %), and Class 4 as “Severe proactive and reactive aggression (no differences between frustration-induced and provocation-induced reactive aggression)”(severe PA & RA) (*N* = 47; 8 %) (Fig. 1). One person was removed from the analysis due to missing values on each of the three factors.

**Table 3:** Fit indices of the Latent Class Analysis (LCA). Values in bold show the best model fit

Amount of classes	BIC	Entropy
2 classes	10,063,02	0.962
3 classes	9840,87	0.920
**4 classes**	**9769,15**	**0.907**
5 classes	9785,04	0.902
6 classes	9789,87	0.879

The four classes show the best fit, since in this model the BIC is the lowest and the Entropy is high

**Fig. 2:**
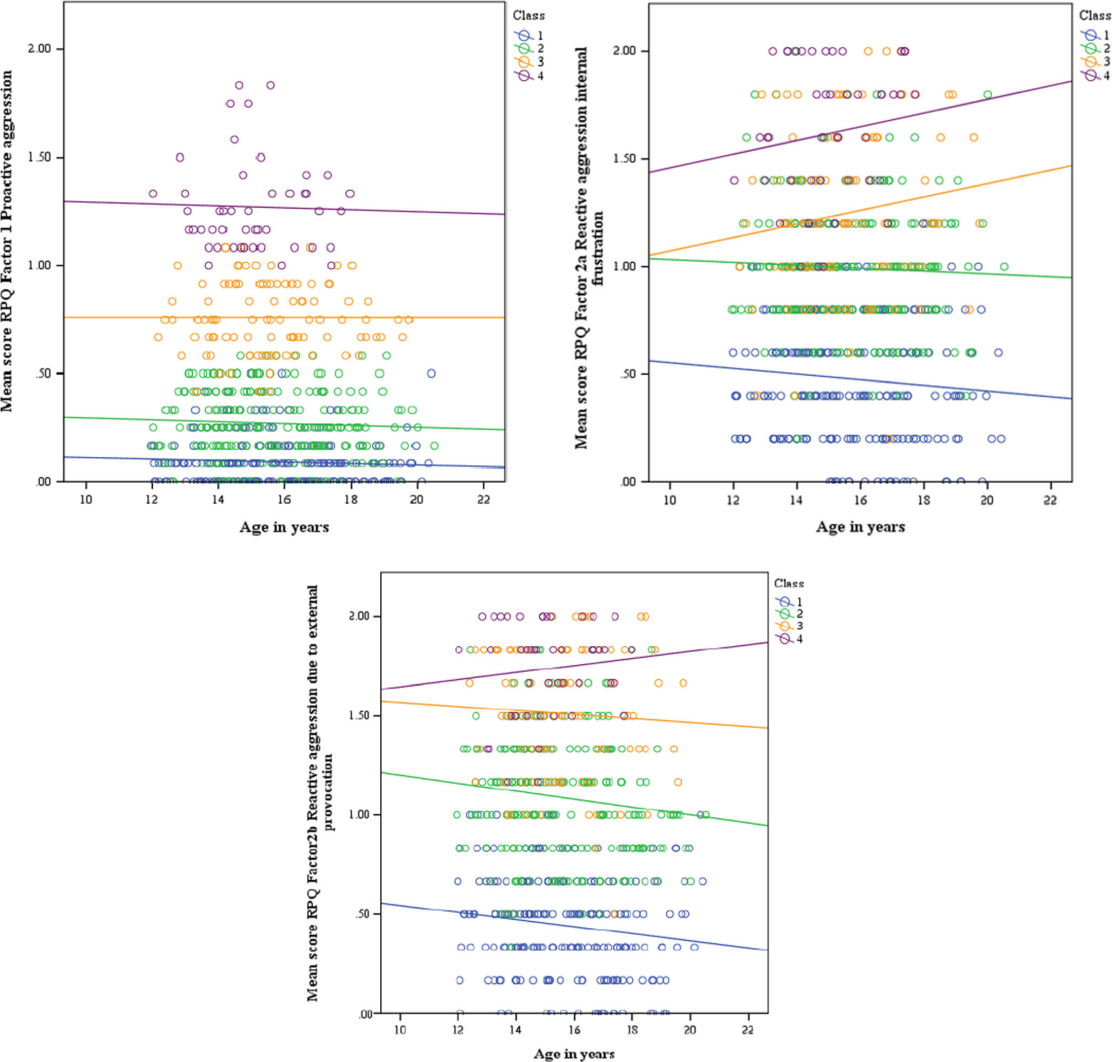
Moderator effect of age by class, per factor

Results were robust across the four different studies from which the data have been aggregated in the multi-level LCA (Supplement S2). These four classes did not differ in age, gender ratio, IQ and ethnicity. All classes had significantly lower scores for proactive than for the two factors of reactive aggression (within-subject analyses; Table 4). Further, class 1 (subclinical aggression) and class 4 (severe PA & RA) showed no significant differences between factor 2 “reactive aggression through internal frustration” and factor 3 “reactive aggression through external provocation” (*p* = 0.18 and *p* = 0.06 respectively), whereas class 2 (Moderate RA) and class 3 (PA & RA) did. The between-class comparison revealed main effects of class for all three aggression factors, with post-hoc tests indicating that scores for all factors were significantly higher in class 4 (severe PA & RA), followed by class 3 (PA & RA), class 2 (RA) and finally class 1 (low levels of aggression) (i.e., class 1 < class 2 < class 3 < class 4; see Table 4). Results were similar when age, gender, IQ and ethnicity were included as covariates in the between-comparison analysis (still a significant main effect of class). There was a small class by gender interaction effect for the factors proactive aggression and reactive aggression due to internal frustration. Post-hoc analyses indicated that boys showed overall higher levels of proactive aggression, and girls showed higher levels of reactive aggression due to internal frustration. However, within class differences were overall very similar for boys and girls (see supplement S4), suggesting gender not to be an important moderator of the individual-based analyses. However, class by age interaction effects were found for all three factors (*p*-values between *p* = 0.002 and *p* = 0.016). Post-hoc analyses indicated that reactive aggression due to external frustration was lowest with older age in all classes (class 1: *r* = −0.14, class 2: *r* = −0.13, class 3: *r* = −0.05) except in the most affected one (class 4: *r* = 0.107). Similarly, reactive aggression due to internal frustration was lowest in older subjects in the two least affected classes (class 1: *r* = −0.09, class 2: *r* = −0.04) but highest in older subjects in the two more severely affected classes (class 3: *r* = 0.14, class 4: *r* = 0.15). These differential age-effects were not substantially present for proactive aggression, showing no substantial relation to age in any of the classes (class 1: *r* = −0.06, class 2: *r* = −0.05; class 3: *r* = <0.01; class 4: *r* = −0.03. As such, important age moderating effects were detected in our re-analyses of the data, suggesting in addition to quantitative (severity) aspects, the four identified classes also appeared to be discriminated by potentially transient/remitting versus aggravating forms of reactive aggression with increasing age (albeit note that our sample was cross-sectional in nature, longitudinal analyses are needed to confirm this observation) (see, Fig. 2). No significant interaction effect of class by IQ (*p* = 0.29) and class by ethnicity (*p* = 0.08) was found (Supplement S4).

**Table 4:** Demographic characteristics of the four classes, derived from the Latent Class Analysis

	Class 1 Low level of aggression *n* = 220	Class 2 Moderate RA *n* = 222	Class 3 RA& PA *n* = 97	Class 4 Severe RA & PA *n* = 47	Significance (*p-*value) of between-class comparison
Age (*M*, *SD*)	15.8 (2.02)	15.6 (1.92)	15.7 (1.74)	15.1 (1.46)	NS *
% Male	73.6 %	73.9 %	68 %	57.4 %	NS
Ethnicity (% non-Caucasian)	53.6 %	48.2 %	51.5 %	57.4 %	NS
IQ (*M*, *SD*)	90.18 (15.65)	87 (15.34)	86.33 (14.86)	89.68 (12.4)	NS
Study % *	1 = 19.8 % 2 = 31.7 % 3 = 51.3 % 4 = 50.5 %	1 = 51.9 % 2 = 33.2 % 3 = 34.4 % 4 = 34.0 %	1 = 21.4 % 2 = 18.6 % 3 = 13.6 % 4 = 10.7 %	1 = 6.9 % 2 = 16.1 % 3 = 0.6 % 4 = 4.9 %	*p* < 0.001
Between class comparisons (weighted mean scores)**					
F1: RPQ proactive	0.09 (0.11)	0.27 (0.15)	0.76 (0.16)	1.27 (0.21)	*p* < 0.001;1 < 2 < 3 < 4^***^
F2: RPQ reactive internal frustration	0.48 (0.28)	0.99 (0.31)	1.25 (0.38)	1.62 (0.32)	*p* < 0.001; 1 < 2 < 3 < 4^***^
F3: RPQ reactive external provocation	0.44 (0.25)	1.08 (0.30)	1.51 (0.32)	1.73 (0.24)	*p* < 0.001; 1 < 2 < 3 < 4^***^
Within class comparisons					
F1 vs. F2	*p* < 0.001	*p* < 0.001	*p* < 0.001	*p* < 0.001	
F2 vs. F3	NS	*p* < 0.001	*p* < 0.001	NS	
F1 vs. F3	*p* < 0.001	*p* < 0.001	*p* < 0.001	*p* < 0.001	

*NS=Not significant. Study: 1=School in Rotterdam; 2=Closed youth care facility 3=committed crime before the age of 12 and 4=delinquent diversion program

**Weighted mean score = mean of the RPQ factor/total items of the scale

***Also when corrected for age, gender, IQ and ethnicity

### Correlations

We assessed the correlation of the three RPQ factors with DSM-based behavioral scales, and broad band internalizing, externalizing and total problem behavior scale scores of the YSR and CBCL. The three RPQ factors were significantly correlated with almost all YSR and CBCL scores. As expected, the strongest correlations (*r*’s > 0.50) were found between the three RPQ aggression factors and total externalizing behavior problems and the ODD, CD and ADHD problem scales. The overall patterns of correlations were very similar for the three RPQ aggression factors (Table 5). However, some support for differential relationships was found using the test of difference between different correlations. Proactive aggression was significantly stronger correlated with lower levels of internalizing problems and higher levels of CD problems than the two reactive scales; both reactive aggression scales were significantly stronger correlated with ADHD compared to the proactive aggression scale, and “reactive aggression due to internal frustration” was significantly stronger correlated with anxiety problems (YSR not CBCL) than proactive aggression and reactive due to external frustration (see Table 5 for details).

**Table 5:** Correlations between the three RPQ factors and the DSM scales of the CBCL and YSR questionnaires

YSR DSM scales	YSR Affective	YSR Anxiety	YSR Somatic	YSR ADHD	YSR ODD	YSR CD	YSR internalizing	YSR externalizing	YSR Total
F1:									
*r*	0.270	0.134^#^	0.159	0.351^#^	0.505	0.659^#+^	0.280^#+^	0.606	0.503
*p-*value	<0.001	0.005	0.001	<0.001	<0.001	<0.001	<0.001	<0.001	<0.001
F2:									
*r*	0.331	0.266^#, ^^	0.214	0.460^#^	0.508	0.503^#^^	0.414^#^	0.587	0.551
*p-*value	<0.001	<0.001	<0.001	<0.001	<0.001	<0.001	<0.001	<0.001	<0.001
F3:									
*r*	0.279	0.176 ^^^	0.183	0.403	0.514	0.589^+^^	0.370^+^	0.608	0.550
*p-*value	<0.001	<0.001	<0.001	<0.001	<0.001	<0.001	<0.001	<0.001	<0.001
CBCL DSM scales	CBCL Affective	CBCL Anxiety	CBCL Somatic	CBCL ADHD	CBCL ODD	CBCL CD	CBCL internalizing	CBCL externalizing	CBCL Total
F1:									
*r*	0.208	0.162	0.029	0.333	0.361	0.425^#,^	0.194	0.435	0.345
*p-*value	<0.001	0.003	NS*	<0.001	<0.001	<0.001	0.001	<0.001	<0.001
F2:									
*r*	0.257	0.184	0.082	0.323	0.311	0.329^#,^	0.240	0.393	0.318
*p-*value	<0.001	0.001	NS	<0.001	<0.001	<0.001	<0.001	<0.001	<0.001
F3:									
*r*	0.266	0.164	0.046	0.306	0.362	0.387	0.198	0.441	0.351
*p-*value	<0.001	0.002	NS	<0.001	<0.001	<0.001	<0.001	<0.001	<0.001

F1=Proactive aggression

F2=Reactive aggression due to internal frustration

F3=Reactive aggression due to external provocation

*NS=Not significant

**#**=F1 ≠ F2; **^** =F2 ≠ F3; **+** = F1≠ F3(2-tailed *p*<0.05)

### Behavioral Profiles of the Indicated Classes

Significant main class effects were found for all of the DSM related CBCL and YSR scales (except for CBCL somatic problems). Post-hoc analysis revealed that, in most cases, problem scores were significantly lower in class 1 (“low aggression”) compared to the other classes (Table 6 and Supplement S3).

**Table 6:** CBCL and YSR subscale mean score and standard deviation (SD) of the different classes

	Class 1 (“Low”)	Class 2 (“Moderate RA”)	Class 3 (“RA&PA”)	Class 4 (“severe RA&PA”)	*p*-values
YSR DSM scales					
YSR affective	*5*4.10 (6.48)	56.77 (7.41)	59.48 (9.01)	60.79 (8.91)	F(3446)=13,69; *p* <0.01; 2=3, 3=4; 1<2<4; 1<3; ^*ƞ*p2^=0.084
YSR anxiety	52.02 (4.12)	53.59 (5.78)	54.58 (6.46)	54.27 (4.50)	F(3432)=5,18; *p* =0.002; 2=3=4; 1=4; 1<2; 1<3; ^*ƞ*p2^ =0.035
YSR somatic	54.11 (6.72)	56.61 (8.38)	58.14 (9.43)	58.14 (9.49)	F(3430)=5,75; *p*=0.001; 2=3=4; 1<2; 1<4; 1<3; ^*ƞ*p2^ =0.038
YSR ADHD	52.72 (4.63)	*5*7.09 (6.70)	59.98 (7.89)	60.26 (8.43)	F(3344)=30,62; *p*<0.001; 3=4; 1<2<3; 2<4; 1<4; ^*ƞ*p2^ =0.17
YSR ODD	51.89 (3.63)	56.27 (6.55)	60.82 (8.19)	63.58 (8.28)	F(3441)=57,18; *p*<0.001; 3=4; 1<2<3; 2<4; 1<4;^*ƞ*p2^ =0.28
YSR CD	53.60 (4.85)	58.08 (6.67)	67.34 (9.12)	71.02 (10.29)	F(3437)=105,09; *p*<0.001; 3=4; 1<2<3; 2<4; 1<4; ^*ƞ*p2^ =0.41
YSR internalizing	44.46 (10.07)	49.94 (10.42)	53.77 (10.45)	54.43 (8.23)	F (3412)=18,92; *p*<0.001; 2=3=4; 1<2; 1<3; 1<4; ^*ƞ*p2^ =0.121
YSR externalizing	47.53 (8.85)	56.23 (9.18)	65.52 (10.42)	69.82 (8.65)	F(3410)= 88.69; *p*<0.001; 1<2<3; 1<2<4; 3=4; ^*ƞ*p2^ =0.394
CBCL DSM scales					
CBCL affective	57.49 (7.75)	60.74 (8.60)	62.50 (8.84)	63.17 (9.47)	F (3333)=7.36; *p*<0.001; 2=3=4; 1<2; 1<3; 1<4; ^*ƞ*p2=^0.062
CBCL anxiety	54.76 (6.52)	57.22 (7.49)	58.33 (7.71)	58.03 (6.16)	F(3338)=4.94; *p*=0.002; 2=3=4; 1=4;1<2; 1<3; ^*ƞ*p2=^0.042
CBCL somatic	56.57 (8.48)	56.75(9.19)	56.96 (8.41)	57.69 (9.18)	F(3344)=0.15, *p*=0.927
CBCL ADHD	55.93 (7.35)	59.02 (7.74)	62.11 (7.17)	63.88 (8.14)	F(3342)=15.32; *p*<0.001; 2=3; 3=4; 1<2<4; 1<3;^*ƞ*p2=^0.118
CBCL ODD	55.31 (7.05)	58.67(7.62)	63.29 (8.22)	63.74 (7.75)	F=(3339)=20,69;*p*<0.001; 1<2<3;1<2<4;3=4;^*ƞ*p2=^0.155
CBCL CD	57.10 (8.17)	60.24 (7.66)	66.62 (9.12)	68.57 (10.29)	F(3320)=27,46; *p*<0.001; 1<2<3;1<2<4;3=4;^*ƞ*p2=^0.205
CBCL internalizing	52.008 (10.73)	55.79 (10.85)	57.84 (9.52)	58.72 (9.00)	F (3285) = 5.45; *p*=0.001; 2=3=4; 1<2; 1<3; 1<4; ^*ƞ*p2^=0.054
CBCL externalizing	52.47 (12.47)	59.90 (10.16)	65.97 (10.32)	70.57 (7.09)	F (3289)=27,98; *p*<0.001; 1<2<3; 1<2<4; 3=4;^*ƞ*p2^=0.225

*ƞ*p 2=partial effect size

Overall, the two most severe classes (“PA & RA” and “Severe PA & RA”) showed significantly higher scores on the ADHD, ODD and CD scales of the YSR and CBCL, compared to the other classes (Table 6). Clinical severity levels were only reached by the “severe PA & RA” class on the CD scale, while the “PA & RA” class reached a subclinical CD symptom level (higher provocation-induced aggression compared to frustration-induced aggression). The average score on the ADHD and ODD scales did not reach a (sub) clinical level threshold in any of the classes. The results of the total problem scales indicate that both proactive and reactive classes showed significantly higher, and (sub)clinical levels of externalizing problem behavior (Table 6). The internalizing behavior problems scale did not reach (sub)clinical levels in any of the classes but was higher in the aggressive classes compared to the low level of aggression. The total problem scale did not significantly differ between classes. Thus, in contrast to our prediction, there were no differential associations between the latent classes and scores on the YSR and CBCL questionnaires. In fact, all association between classes and scores on YSR and CBCL reflected a severity gradient from class 1 up to class 4. In addition, multiple regression analyses were conducted to examine possible suppression relationships. Results showed that in comparison to the zero-order correlations, where reactive and proactive aggression tended to relate similarly to internalizing and externalizing symptoms, weaker relationships were found when reactive and proactive forms of aggression were analyzed simultaneously in relation to externalizing and internalizing symptoms. This underlines the high interrelatedness of the three factors. Of interest was that “reactive aggression due to internal frustration” was the strongest predictor of CBCL and YSR internalizing problems. Furthermore, externalizing problems were predicted by both proactive and reactive aggression due to external provocation, see Table 7.

**Table 7:** Multivariate regression analysis (forward-methods), contribution of three factors in one model

	YSR				CBCL			
	Internalizing		Externalizing		Internalizing		Externalizing	
	Forward^#^	Zero-order	Forward	Zero-order	Forward	Zero-order	Forward	Zero-order
Proactive aggression (Factor 1)	NS	0.28**	0.36**	.61**	NS	0.19**	0.26*	0.44**
Reactive aggression internal frustration (Factor 2a)	0.30**	0.41**	24**	.59**	0.19*	0.24**	NS	0.39**
Reactive aggression external provocation (Factor 2b)	0.16**	0.37**	0.20**	.61**	NS	0.20**	0.23**	0.44**
Shared variance (Total model R-square)	0.24		0.53		0.08		0.29	
Significant F-change *p*-value	0.024		<0.001		0.006		0.007	

*NS*=Not significant

**p<0.001

*p<0.005

^#^Multivariate regression analysis, forward methods with all three factors as predictors taken together

## Discussion

This study was designed to examine whether proactive and reactive aggression are meaningful distinctions at the variable- and person-based level, and to determine their associated behavioral profiles. These aims were examined in 587 adolescents (mean age 15.6; 71.6 % male) from clinical samples of four different sites. The variable-based approach (factor analyses) yielded a three factor solution that was robust across the four different recruitment sites, consisting of *proactive aggression* and *two* forms of reactive aggression: *reactive aggression due to internal frustration* and *reactive aggression due to external provocation*. Proactive aggression was significantly correlated with lower levels of internalizing problems and higher levels of conduct problems; and “reactive aggression due to internal frustration” was significantly stronger correlated with anxiety problems and ADHD problems. Also, internalizing problems rated by parents were uniquely predicted by “reactive aggression due to internal frustration” and self-reported internalizing problems predicted by both subtypes of reactive aggression. However, results showed moderate to high overlap between all three factors. Also, despite the finding that on a variable-based level three different types of aggression seem to be distinguished, the person-based approach (multi-level LCA) identified four classes that mainly differed quantitatively (no “proactive-only” class present), yet also qualitatively when age was taken into account, with reactive aggression becoming more severe with age in the highest affected class yet diminishing with age in the other classes. No proactive-only group could be determined, suggesting that proactive aggression does not exist without reactive aggression or that adolescents with proactive-only aggression are not being referred to clinical practice.

The main findings of the variable-based approach showed that proactive and reactive aggression can be distinguished. However, in line with a recent review of Blair (2013) and study of Fite et al. (2006), our factor model favored a *three* factor solution instead of the expected two factor solution, with “a proactive factor”, a factor “reactive aggression due to internal frustration” and a factor “reactive aggression due to external provocation”. These three factors thus reflected distinguishable but moderate correlated aspects of aggression. Also, the factor “reactive aggression due to external provocation” only revealed three unique items, however this detracts not from the finding that the 3-factor solution described presents the best and justified factor solution. Moreover, a similar three factor model of Blair (2013) was based on neurobiological data to differentiate between “proactive aggression” and “frustration-based reactive aggression”, putatively linked to decreased striatal and ventromedial prefrontal cortex responsiveness, and “threat-based reactive aggression”, associated with increased amygdala responsiveness (Blair 2013). “Frustration-based aggression” is supposed to partly arise as a consequence of inflexibility to changes in the environment, impairments in decision making and is linked to psychopathy and callous-unemotional traits (CU-traits), which seems to correspond with our factor “reactive aggression due to internal frustration”. Furthermore, our “reactive aggression due to external provocation” seems to correspond with the “threat-based aggression” (linked to anxiety and social provocation) since several factor items focus on aggression due to threats or provocation by other people. Our data thus support the hypothesis that reactive aggression may be meaningfully distinguished into frustration-based and threat-based aggression.

Further, we hypothesized differential associations between the aggression factors and YSR and CBCL subscales (Table 5). To be more specific, we expected proactive aggression to be associated with increased levels of conduct disorder symptoms. This hypothesis was confirmed. Overall, very similar associations between all the three factors and ODD/CD scores were reported (all *r* > 0.50). However, proactive aggression was significantly stronger correlated with YSR and CBCL conduct disorder problems (CD) than the two reactive forms of aggression. Furthermore, associations between reactive and proactive aggression and anxiety, affective, somatic and total internalizing symptoms were very similar. However, the YSR (but not CBCL) anxiety scale was significantly stronger correlated with “reactive aggression due to internal frustration” compared to the proactive and the “reactive aggression due to external provocation”. This is in line with our hypothesis that reactive aggression is associated with anxiety, but in contrast with the model of Blair (2013) where “threat-based reactive aggression” was associated with anxiety problems. This could be explained by the fact that the items of the YSR and CBCL anxiety scale mainly focus on fear of animals, going to school, being worried and nervous, and do not focus on being anxious because of threats or provocation, causing a low level of anxiety in the present study. Also, similar associations between ADHD and the three different factors were found, but with significant stronger correlations between the YSR ADHD scale (not the CBCL scale) and the “frustration-based” reactive aggression factor, compared to the “proactive factor”. This is in line with previous research showing inhibition and inattention problems within reactive aggression. Furthermore, the model of Blair shows impaired levels of decision making in the “frustration-based” reactive factor, which is also associated with ADHD problems (Luman et al. 2010). Internalizing problems were significantly stronger associated with the two forms of reactive aggression compared to the proactive form of aggression, which is in line with results of a meta-analysis of Card and Little (2006) regarding proactive and reactive aggression in children and adolescents. Multiple regression analyses showed that internalizing problems were uniquely predicted by “reactive aggression due to internal frustration” rated by parents and predicted by both subtypes of reactive aggression on self-report. Externalizing behavior problems were predicted by all three factors on self-report, and by proactive and reactive aggression due to external provocation on parent-report. However, high interrelatedness of the three factors was shown.

The main results of person-based approach (multi-level LCA) revealed four classes that were characterized by different levels of severity, but with some qualitatively differences when age was taken into account. We were unable to find support for our hypothesis that we would identify individuals with predominantly proactive aggression without reactive aggression. No crossing lines (showing high proactive aggression with low reactive aggression or vice versa) were shown; only gradient, parallel lines of severity. Also, results showed that proactive aggression was not present without reactive aggression in the most severe classes. This shows that moderately severe reactive aggression was present without clinically relevant levels of proactive aggression, but also more severe reactive aggression is generally accompanied by proactive aggression. This is in line with previous research of children between 9 and 14 years old (Crapanzano et al. 2010), showing severity-based subgroups of aggressive individuals, and no proactive-only group. The joint presence of proactive and reactive aggression in the same individuals could be explained in part through how aggression was measured. The correlation between reactive and proactive aggression has found to be lower in observation and computer tasks, as compared to studies using (self-report) questionnaires (Polman et al. 2007). Moreover, it is possible that a proactive-only group does exist in population samples, but not in clinical samples, as this subtype may be less overt (Kempes et al. 2005) and hence does not automatically lead to clinical referral or contacts with police or justice. However, proactive and reactive aggression may be more distinguishable in a population sample. In clinical samples with increasing overall severity of aggression the clinical relevance of these subtypes may be less clear*.*

Furthermore, no moderating effect of gender was found, which is in line with a meta-analysis of 51 studies regarding proactive and reactive aggression (Polman et al. 2007) In addition, age moderating effects were found, with the more severe class showing highest severity of reactive aggression in older subjects, whereas the two least affected classes showed lowest levels of reactive aggression in older subjects. No effect of age was found in proactive aggression. This could implicate that the severity level of reactive aggression –but not proactive aggression- may changed over time (but note, our data were cross sectional in nature, longitudinal data are needed to confirm this). A previous study is in line with our findings showing that in 5 to 18 year old psychiatric referred children reactive aggression –but not proactive aggression- was lowest in older subjects. In contrast, a longitudinal study of Barker et al. (2006) showed that reactive aggression and proactive aggression tend to develop similar trajectories in 13–18 year old high-risk boys. This could be explained by the fact that probably younger adolescents with higher levels of proactive aggression were not included in this study and that reactive aggression often appears earlier in life than proactive aggression (Merk et al. 2007). This might indicate that low to moderate levels of reactive aggression in younger adolescents seems to be more “normal” at younger age when coping strategies are still lacking. However, when not diminishing with age this may become more persistent and severe at older age. This suggests that treatment is needed to prevent aggravating levels of aggression in reactive aggression (and co-occurring proactive aggression). Reactive aggression due to internal frustration seems to be even more aggravating over age than reactive aggression due to external provocation. Furthermore, reactive aggression will give more insight in development of aggression over time than proactive aggression, since both types of aggression co-occur at all levels of severity and no age effect of proactive aggression was found. Overall, clinicians should take age and the development of aggression levels into account, since younger adolescents with higher levels of reactive aggression are at risk to develop more severe levels of reactive aggression (in combination with proactive aggression) at older age. However, future research should be done including longitudinal data to replicate this age by class effect.

We hypothesized differential associations on the person-based approach between aggression classes and internalizing and externalizing scores of the YSR and CBCL (Table 6). Classes with more severe proactive (and reactive) aggression showed higher scores on the ADHD, ODD, CD, and externalizing scales of YSR and CBCL, but this appeared to be driven by overall severity of aggression. Furthermore, no clinically relevant anxiety was found in any of the latent classes.

This study had some limitations. First of all, the RPQ is a self-report questionnaire and therefore answers could be biased or social desirable. However, observation methods, teacher questionnaires or computer task can be biased as well (Polman et al. 2007), therefore a combination of both should be used. Future research should include results of multiple informants and assessments to prevent this bias. Furthermore, we did not use a population sample, which could lead to selection bias and an incomplete sample where possible subgroups (proactive-only) have been left out. Moreover, more boys were included than girls and the YSR and CBCL data were not complete for every group that was included in this study. Also, this study only included “function” of aggression (proactive vs reactive), but not “form” of aggression (i.e., physical or relational aggression) which has been distinguished in previous research (Marsee et al. 2014). Future research should include both forms and functions of aggression and more girls in studies regarding aggression problems or conduct disorder problems. In addition, this study used cross-sectional data and no longitudinal data. Finally, our data-base did not include contextual information, which is information about whether and which environmental triggers and cues elicited aggression in our participants.

Overall, the variable-based analyses demonstrate that proactive and reactive aggression can be distinguished. In fact, three distinguishable but strongly correlated factors of aggression were identified. The original proactive factor and reactive aggression was divided into two different forms; “reactive aggression due to internal frustration” and “reactive aggression due to provocation”. These three forms of aggression show, besides similar and overlapping behavioral associations, also some specific associations; namely lower associations with internalizing problems and higher associations with CD in proactive aggression; higher associations of anxiety, ADHD and internalizing problems were found in the “reactive aggression due to internal frustration”.

However, despite the fact that proactive and reactive aggression can be distinguished at the variable-based level, the clinical relevance of these findings is challenged by the person-based analysis showing proactive and reactive aggression are mainly driven by aggression severity. If proactive aggression is present (in combination with reactive aggression), clinical levels of conduct disorder and externalizing behavior problems are reported. This suggests the presence of proactive aggression can be seen as a severity marker that need extra awareness of clinicians. Also, age effects are important to take into account in clinical practice. Findings suggest that reactive aggression is a more “normal” phenomenon at younger age and when not diminishing with age it may be a marker for the most severe aggression in older adolescents. Although it seems reasonable that subjects showing high levels of proactive and reactive aggression, and younger adolescents who are at risk of developing more severe reactive aggression warrant more intensive respectively preventative treatment than those showing reactive aggression only, future research should address the question of differential responsivity to treatment (Vitaro et al. 2006). Future research should focus on the differentiating and/or overlapping neurocognitive (i.e., impaired decision making), neural, biologic, behavioral (CU-traits, trauma) and genetic profiles of the three different aggression factors (proactive, frustration-induced and threat-induced). This would enable to explore whether a distinction of aggression based on these profiles would produce a stronger differentiation than a distinction based on observable behaviors.

## Electronic supplementary material

ESM 1 (DOCX 84 kb)

ESM 2 (DOCX 84 kb)

ESM 3 (DOCX 81 kb)

ESM 4 (DOCX 39 kb)
