# Supplementary material for: Are Proactive and Reactive Aggression Meaningful Distinctions in Adolescents? A Variable- and Person-Based Approach
Source: J Abnorm Child Psychol. 2016 Apr 26;45(1):1–14. doi: 10.1007/s10802-016-0149-5 (PMC5219021; doi:10.1007/s10802-016-0149-5)
Supplement: Supplementary file 1 — (DOCX 84 kb) [file 10802_2016_149_MOESM1_ESM.docx]

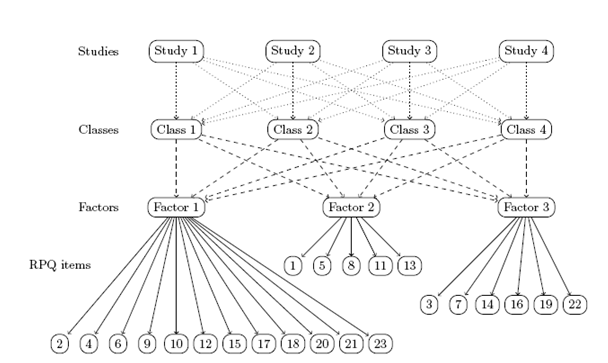
Supplement 1: Model used in the multi-level Latent Class Analysis. The model shows the 23-items that underlie the 3 factors that were found in the EFA (straight lines). The factor loadings are shown in Table 2. Furthermore, the model shows the four different classes that were revealed from the LCA and their relation (dashed lines) with the three different factors (see Figure 1). The model corrected for within-center measurement bias, by taking the different studies into account (dotted lines). See Table 3 for the distribution of the studies among the revealed classes.
