# Supplementary material for: Are Proactive and Reactive Aggression Meaningful Distinctions in Adolescents? A Variable- and Person-Based Approach
Source: J Abnorm Child Psychol. 2016 Apr 26;45(1):1–14. doi: 10.1007/s10802-016-0149-5 (PMC5219021; doi:10.1007/s10802-016-0149-5)

Supplement 2: a) Results of the LCA, combining the four different studies, b) results of the LCA showing the different studies separately per class.


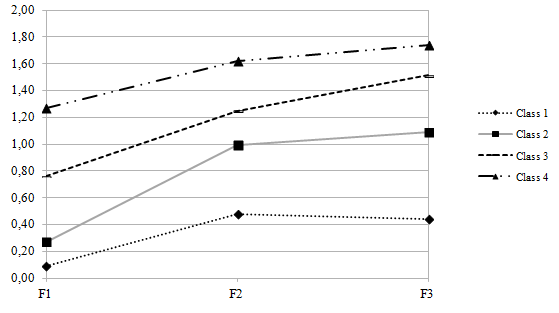
a)

b)
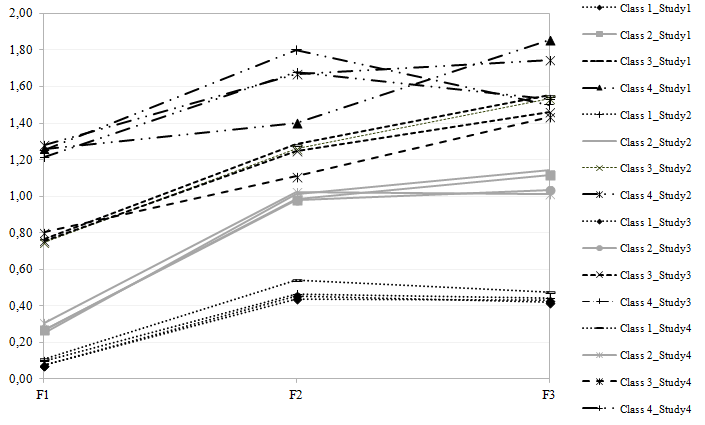

Supplement: Supplementary file 2 — (DOCX 84 kb) [file 10802_2016_149_MOESM2_ESM.docx]
