# Supplementary material for: Are Proactive and Reactive Aggression Meaningful Distinctions in Adolescents? A Variable- and Person-Based Approach
Source: J Abnorm Child Psychol. 2016 Apr 26;45(1):1–14. doi: 10.1007/s10802-016-0149-5 (PMC5219021; doi:10.1007/s10802-016-0149-5)
Supplement: Supplementary file 3 — (DOCX 81 kb) [file 10802_2016_149_MOESM3_ESM.docx]

Supplement 3: The underlying behavior scales per different class. a) YSR DSM scales, b) CBCL DSM scales, c and d) YSR and CBCL internalizing and externalizing scales. The cut-off scores of the subclinical and clinical ranges are displayed in the figures. The error bars represent 1 standard error.


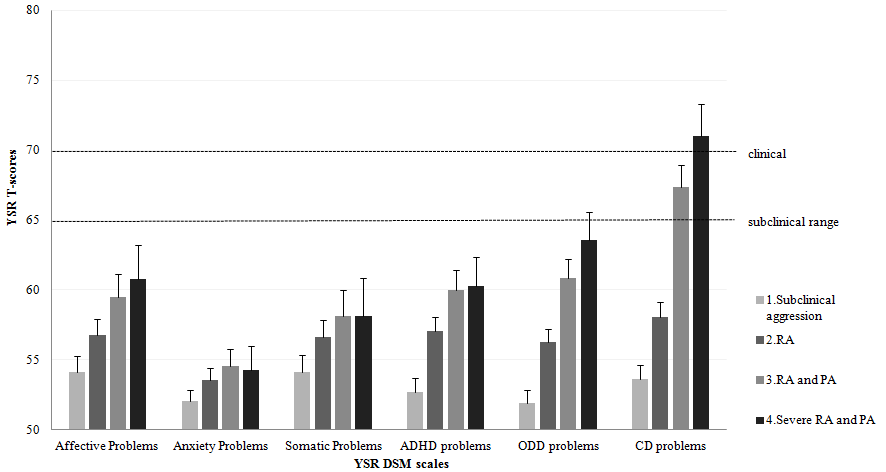


b)


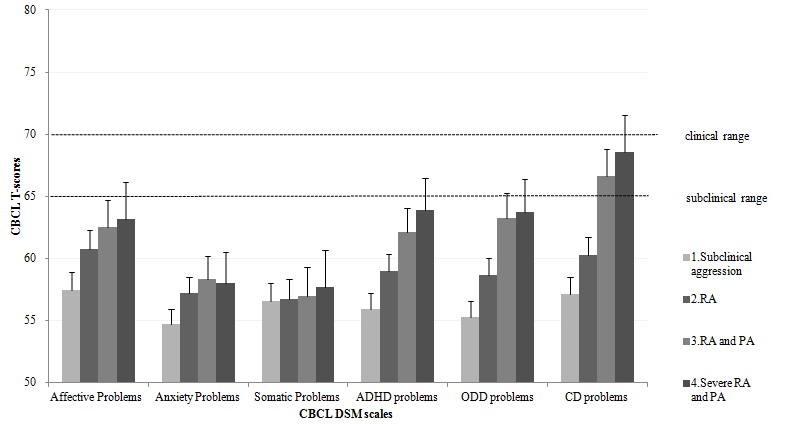


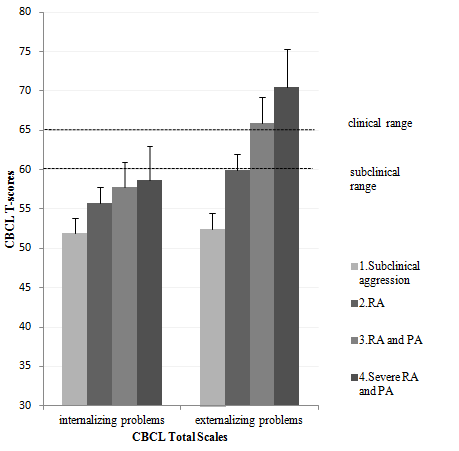

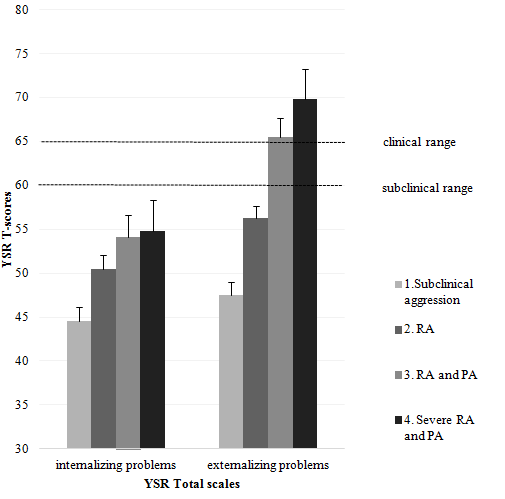
c en d)
