# Supplementary figures and images for: Are Proactive and Reactive Aggression Meaningful Distinctions in Adolescents? A Variable- and Person-Based Approach
Source: J Abnorm Child Psychol. 2016 Apr 26;45(1):1–14. doi: 10.1007/s10802-016-0149-5 (PMC5219021; doi:10.1007/s10802-016-0149-5)

Supplement 4:Class by gender effect


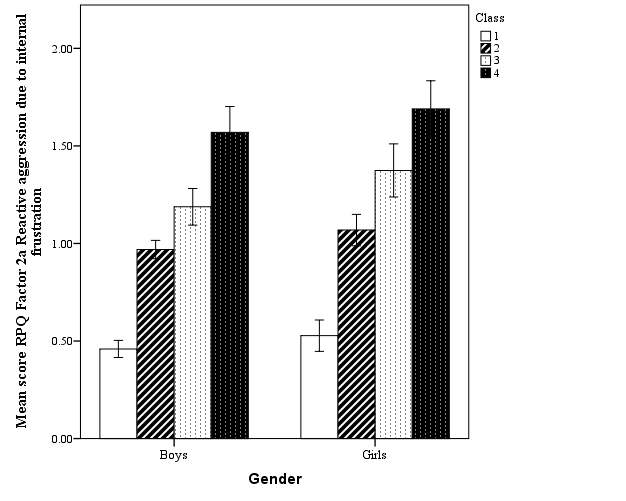


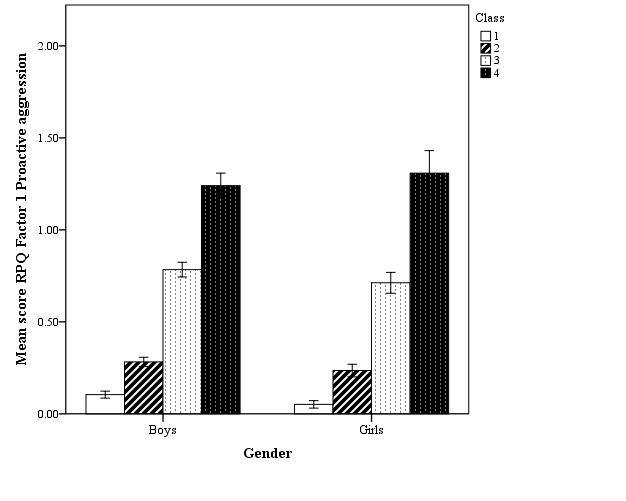


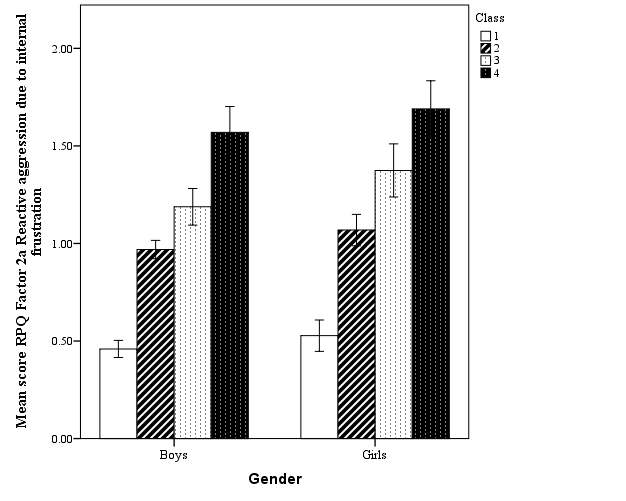

Supplement: Supplementary file 4 — (DOCX 39 kb) [file 10802_2016_149_MOESM4_ESM.docx]
